# A pooled genome-wide screening strategy to identify and rank influenza host restriction factors in cell-based vaccine production platforms

**DOI:** 10.1038/s41598-020-68934-y

**Published:** 2020-07-22

**Authors:** David M. Sharon, Sean Nesdoly, Hsin J. Yang, Jean-François Gélinas, Yu Xia, Sven Ansorge, Amine A. Kamen

**Affiliations:** 1 Department of Bioengineering, McGill University, McConnell Engineering Building, Room 363, 3480 Rue University, Montreal, QC H3A 2K6 Canada; 2 Human Health Therapeutics, National Research Council of Canada, Montreal, QC Canada

**Keywords:** Computational biology and bioinformatics, Gene expression, Expression systems

## Abstract

Cell-derived influenza vaccines provide better protection and a host of other advantages compared to the egg-derived vaccines that currently dominate the market, but their widespread use is hampered by a lack of high yield, low cost production platforms. Identification and knockout of innate immune and metabolic restriction factors within relevant host cell lines used to grow the virus could offer a means to substantially increase vaccine yield. In this paper, we describe and validate a novel genome-wide pooled CRISPR/Cas9 screening strategy that incorporates a reporter virus and a FACS selection step to identify and rank restriction factors in a given vaccine production cell line. Using the HEK-293SF cell line and A/PuertoRico/8/1934 H1N1 influenza as a model, we identify 64 putative influenza restriction factors to direct the creation of high yield knockout cell lines. In addition, gene ontology and protein complex enrichment analysis of this list of putative restriction factors offers broader insights into the primary host cell determinants of viral yield in cell-based vaccine production systems. Overall, this work will advance efforts to address the public health burden posed by influenza.

## Introduction

### Cell-based influenza vaccine production

Mitigating the disease burden of influenza virus is an ongoing public health challenge, with 290,000–650,000 deaths annually and ten-fold as many hospitalizations worldwide^1,2^. The efficacy of influenza vaccines, which must be reformulated annually to adjust for antigenic drift and changes in dominantly circulating strains, has fluctuated between 10 and 60% since 2005^3^.

The vast majority (85–90%) of these vaccines are currently produced using embryonated chicken eggs, but a growing body of evidence suggests significant improvements in vaccine efficacy could be made by switching to cell-based manufacturing platforms^4^. Passaging influenza through eggs induces antigenic drift as the virus adapts to an avian host^4–6^. As a result, egg-based vaccines exhibit a 15–20% decrease in protection rate compared to similarly formulated cell-based vaccines^6–8^. Cell-based platforms can also drastically reduce production lead time by accelerating seed stock reassortment via reverse genetics^9^. This, in turn, reduces the chance of major changes in circulating strains occurring between initial strain selection and vaccine release, an issue that rendered the 2014–2015 seasonal vaccine largely ineffective^10,11^. Additionally, the manufacturing capacity of cell-based vaccines is not constrained by the availability of billions of pathogen-free, synchronously fertilized chicken eggs, which would facilitate rapid response to influenza pandemics if they arise. Other advantages of cell-based vaccines include a lack of allergen contamination and better growth of certain strains^8^.

For all the benefits of cell-based influenza vaccines, current production platforms generally exhibit 4 to 10 fold lower volumetric yield than egg-based counterparts and are 40–100% more expensive^8,12^. Given that influenza vaccine demand exceeds 1.5 billion doses annually, the advantages of cell-based vaccines are irrelevant if high yield, low cost platforms for industrialization are lacking^8^. Past efforts to intensify cell-based vaccine production and drive down cost have largely centered on the optimization of media formulations, infection and harvest parameters, and feeding strategies. However, the advent of CRISPR/Cas9-mediated genome editing technology allows for an additional, relatively unexplored means of increasing vaccine yield: genetic engineering of the host cell line.

### Screen rationale

Cells contain restriction factors that defend against viral infection. Identification and knockout of these restriction factors in host cells could create a more permissive environment for viral replication, thus increasing vaccine yield. However, the use of data from the relevant literature to identify and rank candidate restriction factors is mired by a lack of agreement between the results of independent studies on the gene-to-gene level. For instance, the results of two high-quality genome-wide studies to find influenza restriction factors by Heaton et al. (2017) and Tripathi et al. (2015) are largely consistent in terms of the functional pathways and processes identified^13,14^. However, the overlap between the sets of individual genes identified in the respective studies was less than would be expected by random chance (see Supplemental S1). These results reflect the importance of cell line, culture parameters, influenza strain, and other contextual factors in determining the role which individual genes will impact viral yield. This is particularly true for cell lines used to produce influenza vaccines such as MDCK, Vero, and HEK-293, which diverge heavily from primary cells and each other in terms of their innate antiviral response^12,15^. The question of which gene knockouts will give the greatest increase in vaccine yield is then best probed on a case-by-case basis, using a genome-wide screen conducted on the relevant host cell line.

Wu et al. (2017) and van der Sanden et al. (2016) have attempted to use genome-wide screens to identify viral restriction factors in Vero cells and improve vaccine yield, with variable success^16–18^. In a retrospective analysis, Hoeksema et al. (2018) identified several factors that may explain the difficulties encountered^18^, particularly the issue of using human-specific libraries in non-human derived cell lines. Both screens also used RNAi-based methodologies to probe for restriction factors. Though this is generally more sensitive than CRISPR-based methods, and therefore desirable for basic research, difficulties can be encountered translating the results of RNAi screens into CRISPR/Cas9 knockout cell lines^18^. A further possible source of error is the arrayed format of previous screens. Arrayed screens, in which homogenous knockout populations are physically isolated, can require thousands of microtiter plates and hundreds of thousands of individual manipulations to achieve genome-wide coverage^19^. Aside from being resource intensive to conduct, the inherent difficulties in controlling a screen of this scale tend to result in high variability^19,20^. Alternatively, pooled screening formats, wherein a mixed knockout population is contained in a single vessel, ensure identical treatment of all cells and can achieve genome-wide coverage without high-throughput robotics and other specialized facilities. However, pooled screens are limited in terms of readout, requiring some form of selection that results in enrichment/depletion of knockout populations^20^. This complicates the assessment of phenotypes such as viral yield, which do not directly impact cell survival.

In this study, we present the results of a pooled screening strategy using a reporter virus coupled with a Fluorescence Assisted Cell Sorting (FACS) based selection method to identify and rank host restriction factors for A/Puerto Rico/8/1934 H1N1 (PR/8) influenza in HEK-293SF cells.

## Results

### Screen design

**Figure 1:**
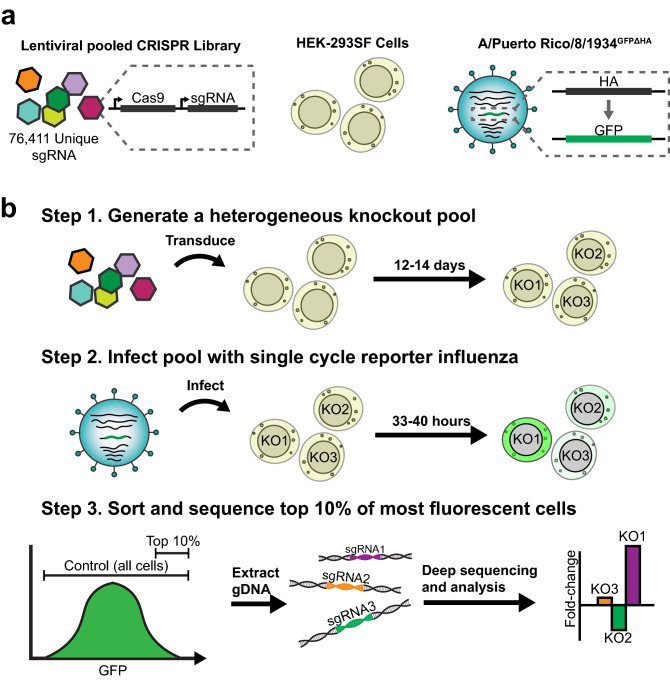
Illustration of pooled screen to identify and rank putative influenza restriction factors. The components of the screen are shown: (**a**) The lentiviral-vectored Brunello Human CRISPR Knockout Pooled Library, consisting of a pool of 76,411 unique sgRNA as well as 1,000 non-targeting controls, each with a CRISPR/Cas9 expression cassette packaged in lentiviral vectors; The HEK-293SF cell line; The A/Puerto Rico/8/1934^GFPΔHA^ (PR/8^GFPΔHA^) reporter virus, wherein the HA coding sequence on genomic segment 4 of the virus has been replaced with the coding sequence of GFP. (**b**) The workflow of the screen is shown, including library transduction, infection with reporter influenza, and selection of a “high yield” knockout population via FACS. The high yield fraction is composed of the top 10% of all GFP expressing cells. A control population, composed of all GFP-expressing cells, is also collected. Deep sequencing is then used to determine the abundance of integrated sgRNA expression cassettes in the genomic DNA (gDNA) of the two fractions. Fold-change enrichment of sgRNA species in the high yield fraction relative to the control is then used to identify and rank putative restriction factors.

The screen components and workflow are described in Fig. 1.

The Brunello Human CRISPR/Cas9 Knockout Pooled Library (Fig. 1a) consists of a pool 76,411 unique sgRNA, each with a CRISPR/Cas9 expression cassette, packaged in lentiviral vectors^21^. This library was selected due to redundant (~ 4×) coverage of all protein coding genes, the inclusion of extensive (n = 1,000) non-targeting controls, and to avoid the confounding effects of using an siRNA library to direct the creation of CRISPR/Cas9 knockout cell lines^21^. The HEK-293SF cell line (Fig. 1a), a serum-free and suspension adapted variant of the HEK-293 parent line, was selected due to its human origin and the availability of a well-annotated genome to ensure minimal off-target effects^22,23^. The HEK-293SF line is also highly attractive from a bioprocess standpoint and, though currently less popular than the Vero or MDCK lines for influenza vaccine production, has shown considerable potential as a scalable and high yield vaccine production platform^9,22,24,25^.

The screen presented in Fig. 1 has been designed to enable a pooled screening format, using a single-cycle reporter virus and a FACS-based selection step. The A/Puerto Rico/8/1934^GFPΔHA^ (PR/8 ^GFPΔHA^) reporter virus (Fig. 1a) can enter cells and replicate wild-type infection kinetics for one infection cycle, but it is unable to produce infectious progeny due to the lack of the HA coding sequence, a critical entry and attachment factor^26^. Furthermore, as we will show in subsequent results, PR/8^GFPΔHA^ transcribes GFP in direct proportion to wildtype PR/8 HA transcription, and GFP expression predicts relative wildtype viral titer. Critically, this allows the individual viral yield of each cell in the knockout pool to be approximated via GFP fluorescence intensity. High yield cells in the knockout pool can then be selected via FACS. The relative abundance of various CRISPR/Cas9 cassettes within the genomic DNA of these cells, integrated via the lentiviral vectors, can then be determined with deep sequencing to identify candidate restriction factors (Fig. 1b). The pooled screening format enables the entire knockout pool of a given replicate to be contained in a single 3L shaker flask, ensuring identical conditions for all cells during the screen. The use of a single-cycle infection virus avoids the confounding effects of secondary infection from neighboring cells.

### PR/8^GFPΔHA^ closely replicates PR/8 infection kinetics and effects on host cells for one infection cycle

**Figure 2:**
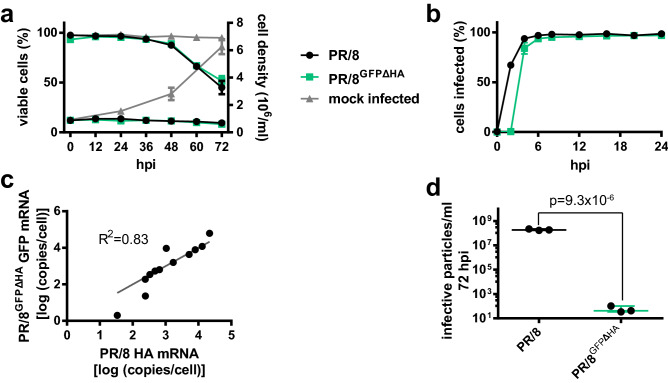
Comparison of PR/8 and PR/8^GFPΔHA^ infection kinetics and effect on cell viability and growth. (**a**) Effect of PR/8 and PR/8^GFPΔHA^ on cell viability and growth compared to mock-infected controls. (**b**) Infection kinetics of PR/8 and PR/8^GFPΔHA^, infected cells are defined as those expressing the influenza nucleoprotein, as measured by flow cytometry. (**c**) Correlation between expression of PR/8 HA mRNA and PR/8^GFPΔHA^ GFP mRNA over the course of a 40 h infection. Cellular mRNA content was measured by strand-specific dPCR. (**d**) Comparison of infective viral particle titer in cell-free supernatant (CFS) of PR/8 and PR/8^GFPΔHA^ infected cultures 72 h hpi. In all experiments, datapoints represent the average of n = 3 independent replicates run in parallel. Error bars represent standard error of the mean (SEM). Whisker plots show mean, range, and individual data points. R^2^ value was determined by linear regression, and *p* value was determined using 2-tailed Student’s *t* test.

The first step before conducting the screen was to investigate how closely PR/8^GFPΔHA^ mirrors the infections kinetics of PR/8 for a single infection cycle. HEK-293SF cells were infected under conditions identical to those used in the screen. Over the next 72 h, samples of cells were assayed for the percentage of cells infected, cell density, and cell viability. In addition, cellular levels of mRNA for genomic segment 4 (HA/GFP) were measured over 40 h in the two cultures to determine whether the modified genomic segment 4 in PR/8^GFPΔHA^ is transcribed at the same rate as that of wildtype PR/8. Results are shown in Fig. 2.

Both PR/8^GFPΔHA^ and PR/8 infected cultures showed identical effects on host cells, inducing immediate cell cycle arrest and declining viability 40 h post-infection (hpi) compared to mock infected controls (Fig. 2a). In terms of infection kinetics, PR/8^GFPΔHA^ cultures showed a 1–2 h delay in viral entry and expression of the influenza nucleoprotein (NP) as assessed by staining and flow cytometry (Fig. 2b). However, within 4 h, virtually all cells in both cultures were infected (Fig. 2b), indicating that PR/8^GFPΔHA^ has no significant defects in attachment and entry compared to wildtype. See Supplementary S2 for flow cytometry gating. There was also a strong linear correlation (R^2^ = 0.83) between the absolute levels of cellular GFP mRNA and HA mRNA expressed over the course of 40 h in PR/8^GFPΔHA^ and PR/8 cultures (Fig. 2c) showing that the modified genomic segment 4 of PR/8^GFPΔHA^ is transcribed at a similar rate as that of PR/8 segment 4 and is an accurate predictor of PR/8 transcription levels. Overall, PR/8^GFPΔHA^ closely replicates PR/8 infection kinetics and effect on host cells under the conditions tested. Finally, we also sought to verify the “single cycle” nature of PR/8^GFPΔHA^ infection. As expected, PR/8^GFPΔHA^ cultures 72hpi contain negligible concentrations of infectious viral particles compared to wildtype PR/8 (Fig. 2d).

### PR/8^GFPΔHA^ GFP reporter intensity predicts PR/8 viral yield

**Figure 3:**
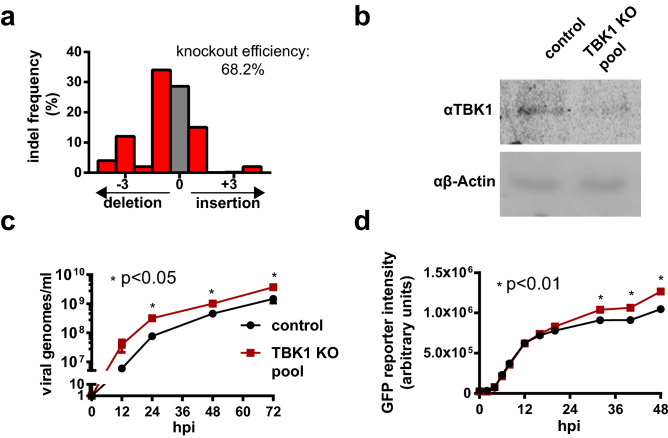
Validation of PR/8^GFPΔHA^ GFP intensity as a reporter for increased viral titer using a HEK-293SF^ΔTBK1^ knockout pool. (**a**) TIDE analysis of gDNA extracted from the HEK-293SF^ΔTBK1^ knockout pool at the sgRNA cut site. Frameshift from control sequence indicates successful indel at the gRNA target site and disruption of the TBK1 gene. Indel frequency is estimated at 68%. (**b**) Western blot for TBK1 against extracts from the HEK-293SF^ΔTBK1^ knockout pool and control cells showing a reduction in TBK1 protein levels in the knockout pool. (**c**) Timecourse measurement of wildtype PR/8 viral titers in terms of genomes/mL in HEK-293SF^ΔTBK1^ and control HEK-293SF CFS. Cells were infected at an MOI of 0.1. Results show significantly elevated viral titers generated by the HEK-293SF^ΔTBK1^ knockout pool. (**d**) Timecourse measurement of GFP reporter intensity in HEK-293SF^ΔTBK1^ and control HEK-293SF cells infected with PR/8^GFPΔHA^. Results show significantly elevated GFP reporter intensity in HEK-293SF^ΔTBK1^ cells. TIDE analysis was conducted using the TIDE webtool. Datapoints of figures (**c**) and (**d**) represent the average of n = 3 independent replicates run in parallel. Error bars represent SEM. All *p* values were determined using using 2-tailed Student’s *t* test. Uncropped Western blot is shown in Supplemental S3.

Though the results of Fig. 2c show that GFP reporter transcription in PR/8^GFPΔHA^ infected cells correlates well with wildtype PR/8 transcription, we wanted to assess whether GFP reporter expression was also indicative of viral yield using a positive control wherein a known restriction factor is knocked-out. Results are shown in Fig. 3.

Tank binding kinase 1 (TBK1) is a serine-threonine kinase and a key component of the retinoic acid-inducible gene I (RIG-I) dsRNA sensing pathway^27^. Disruption of TBK1 expression has been shown to increase PR/8 titer in HEK-293^27,28^. In order to examine whether GFP expression in PR/8^GFPΔHA^ infected cells could predict increases in wildtype viral yield, CRISPR/Cas9 was used to knock out TBK1 in HEK-293SF cells, generating a HEK-293SF^ΔTBK1^ knockout pool. Tracking of Indels by Decomposition (TIDE) analysis (Fig. 3a) and Western blot (Fig. 3b) show successful modification at the target DNA loci and a reduction in TBK1 protein levels in the HEK-293SF^ΔTBK1^ pool, respectively. Wildtype PR/8 yield was then assessed in these cells. As shown in Fig. 3c, cells from the HEK-293SF^ΔTBK1^ knockout pool showed a significant increase in viral yield compared to controls. Both unmodified cells and those from the HEK-293SF^ΔTBK1^ knockout pool were then infected with PR/8^GFPΔHA^, and the mean GFP fluorescence intensity of infected cells was measured over 48 h via flow cytometry. The flow cytometry gating strategy is depicted in Supplemental Figure S2. As shown in Fig. 3d, the mean GFP intensity of the HEK-293SF^ΔTBK1^ knockout pool was significantly elevated relative to control cells. This demonstrates that high yield knockout populations can be identified by GFP intensity from PR/8^GFPΔHA^ reporter virus infection, validating its use in the screen.

### Screen controls and quality metrics performed as expected

**Figure 4:**
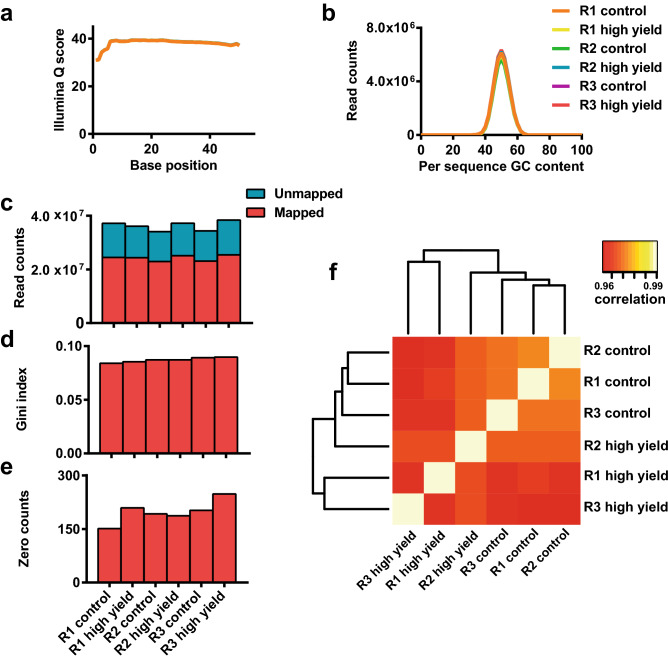
Deep sequencing and read mapping quality metrics. Six samples, consisting of high yield and control fraction for each of the three replicates (abbreviated R1, R2, and R3), were sequenced together as a barcoded pool. (**a**) Average per base Illumina Q score, an aggregate measure of base-calling quality, for each sample. (**b**) Distribution of average GC content per read for each sample. (**c**) Illustration of total reads per sample and reads that were successfully mapped to the library. (**d**) Gini index for each sample, a measure of read distribution across the library. (**e**) Number of zero counts, elements in the library for which no read was mapped, for each sample. (**f**) Heat map showing sample grouping and correlations between samples. Data was generated using the MAGeCK software suite.

Three independent replicates of the screen depicted in Fig. 1 were run, each generating a control (all infected cells) and high yield fraction (cells in the 90th percentile of GFP expression), totaling six samples. FACS gating strategy for sample collection is shown in Supplemental Figure S2. Figure 4 shows assessment of screen replicates in terms of deep sequencing base calling quality, read mapping, and the distribution of these reads within the library.

For all samples, average per base Illumina Q score, an aggregate measure of base calling quality, was > 30 for the entirety of the 50 bp read (Fig. 4a), indicating good base calling during sequencing. GC content per read was consistent between samples and indistinguishable from the theoretical distribution for the library (Fig. 4b), indicating that there was no significant bias in the PCR used to generate the amplicon libraries or in the deep sequencing itself. For all samples, > 65% of reads were mapped to the library (Fig. 4c), further indicating satisfactory sequencing quality. The library was also well represented in terms of both average read depth (~ 300 per sgRNA) (Fig. 4c), and a Gini index, a measure of read distribution across the library, below 0.1 for all samples. Zero count sgRNAs, elements in the library for which no read was mapped, comprise < 1% of total library elements (n = 76,411) for all samples (Fig. 4e). Figure 4f shows correlation and clustering between samples. As expected, the strongest correlations are seen between the three control samples and between the three high yield fractions, respectively.

**Figure 5:**
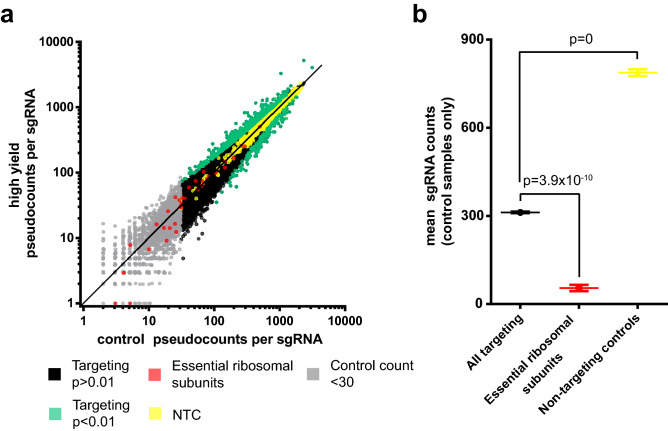
sgRNA abundance, enrichment, and controls. (**a**) Log–Log plot of abundance for each individual sgRNA in high yield and control samples. Values represent the median of three replicates. A pseudocount of + 1 was added to all sgRNA to allow plotting of zero count sgRNA. Elevation above or below the line x = y indicates enrichment or depletion of that sgRNA, respectively. Significantly enriched or depleted sgRNA (n = 754), non-targeting control (NTC) sgRNA (n = 1,000), and controls in the form of sgRNA that target essential ribosomal subunits (n = 34) are highlighted. sgRNA with a median read count in control samples of < 30 (n = 1,760) are displayed but were excluded from downstream analysis due to insufficient representation. (**b**) Mean abundance in control samples of all targeting sgRNA, sgRNA targeting essential ribosomal subunits, and NTC sgRNA. Significant enrichment/depletion of individual sgRNAs was determined using the MAGeCK software suite. Note that *p* values have been adjusted for multiple testing using the Benjamini–Hochberg procedure. Significance values in plot (**b**) were determined using 2-tailed Student’s *t* test. Whisker plots represent mean and SEM.

The library contains an average of four distinct sgRNA targeting towards each protein coding gene. Figure 5a shows the median abundance and enrichment of sgRNA reads for the three replicates.

A total of n = 754 sgRNA were significantly enriched or depleted with a threshold of *p* < 0.01 in high yield fractions. As shown in Fig. 5b, the mean count of all sgRNA targeting essential ribosomal subunits (as identified by Hart et al. (2014)) is significantly lower (*p* = 3.9 × 10^−10^) than the mean of all other targeting sgRNA in control samples^29^. The depletion of this subpopulation in which essential cell survival factors have been targeted for knockout indicates efficient CRISPR/Cas9 activity in the screen. Non-targeting controls were found to be significantly elevated compared to targeting sgRNA, which is an expected result considering we observe a 24–48 h growth arrest in HEK-293SF following CRISPR/Cas9 knockout. We speculate this is due to cells resolving the DNA damage that mediates CRISPR/Cas9 activity, as has been reported elsewhere^30^. See Supplemental File S4 for sgRNA count data.

### Identification and analysis of putative influenza restriction factors in HEK-293SF

**Figure 6:**
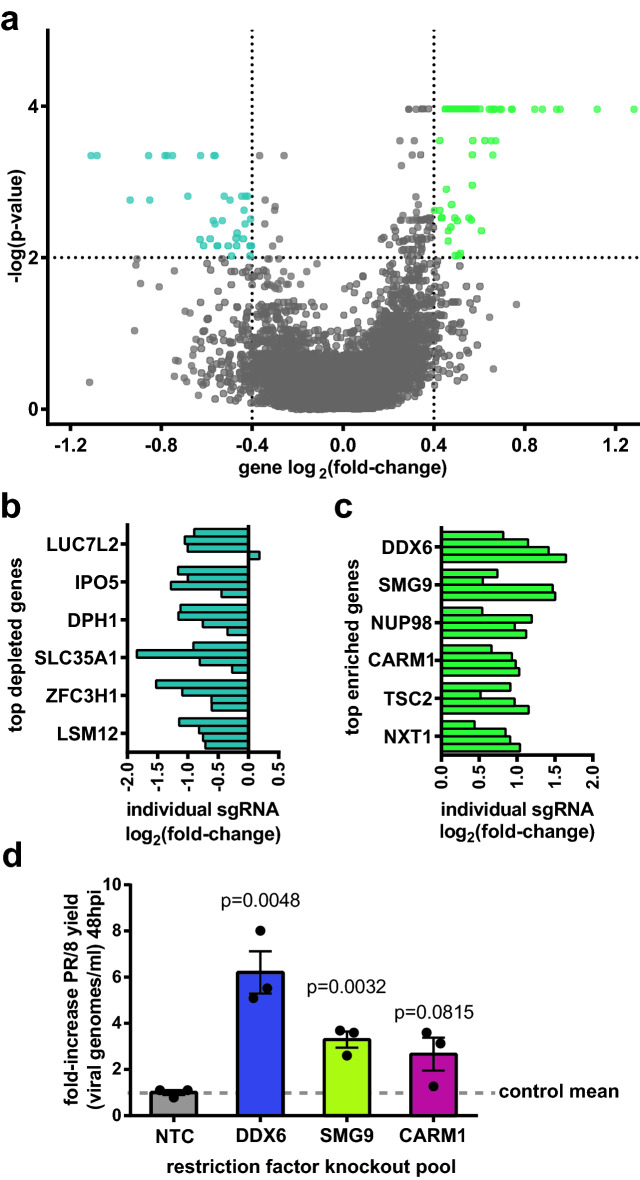
Summary of significantly enriched and depleted genes and hit validation. (**a**) Volcano plot showing the mean log_2_ fold enrichment (lfc) and *p* value for each gene knockout. Gene “hits” are defined as having *p* < 0.01 and |lfc|> 0.4. Using these criteria, n = 64 putative restriction factors were identified, as well as n = 37 significantly depleted genes. (**b**) Individual sgRNA lfc for the six most depleted genes in the screen, i.e. knockouts which were detrimental to influenza replications. (**c**) Enrichment of individual sgRNA corresponding to the top six putative restriction factors identified in the screen. (**d**) Validation of top putative restriction factors DDX6, SMG9, and CARM1 using wildtype PR/8 virus at an MOI of 0.1. Results indicate a 3 to 6–fold increase in viral titer at 48hpi following restriction factor knockout. Significance values in plot (**d**) were determined using 2-tailed Student’s *t* test, with n = 3 independent replicates run in parallel. Error bars represent SEM. Values in panels (**a**–**c**) were obtained by Robust Rank Aggregation (RRA) analysis with the MAGeCKFlute software suite and plotted in Prism. Note that *p* values have been adjusted for multiple testing using the Benjamini–Hochberg procedure. See methods section for analysis parameters.

A total of n = 135 significantly enriched gene knockouts were identified by Robust Rank Aggregation (RRA) analysis using the MAGeCKFlute software suite and a significance threshold of *p* < 0.01^31^. See methods section for analysis parameters. An arbitrary threshold for minimal biological significance of log_2_ fold-change (lfc) > 0.4 was also imposed to refine the results. Overall, n = 64 gene knockouts were identified with these criteria, hereafter referred to as putative restriction factors (Fig. 6a). Although not a goal of this study, we also identified a subset of genes that were significantly depleted that may be of interest, particularly to those looking to identify host targets of antiviral drugs (Fig. 6b). However, we caution that the screen lacks adequate controls to distinguish whether these depleted factors exert a specific effect on influenza replication, or merely that their knockout is detrimental to the cell’s overall biosynthetic capacity. Top putative restriction factors in the screen are shown in Fig. 6c. The full output of the MAGeCKFlute analysis, including a list of all genes and their associated RRA score, lfc, and *p* value, is available in Supplementary data file S4. In addition, because this screen used an indirect readout (GFP florescence) to assess viral titer’ it was important to validate a portion of the putative restriction factors identified by direct measurement of  wildtype PR/8 titers to verify the integrity of the dataset. For each of the genes DDX6, SMG9, and CARM1, a knockout pool was generated using a randomly selected sgRNA sequence from the Brunello Library. Editing efficiency was estimated by TIDE analysis as > 80% for all pools (see Supplemental S5). Cells were then infected with PR/8, and fold-change in influenza yield measured compared to cells transduced with a non-targeting control sgRNA (Fig. 6d). Results show an increase of viral titer of three–sixfold over non-targeting controls, verifying the results of the screen.

**Figure 7:**
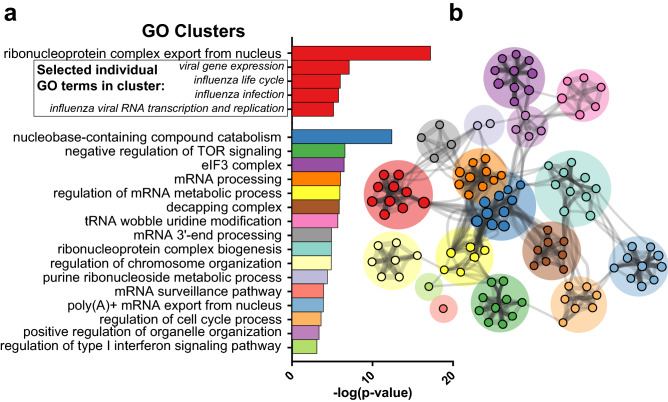
Gene ontology (GO) analysis of putative restriction factors. (**a**) Significantly enriched GO clusters obtained from the 64 putative restriction factors in the screen. In total, n = 274 individual GO terms were found to be enriched with a threshold of *p* < 0.01. Associated GO terms were clustered, with the name of that cluster represented by the individual GO term with the lowest *p* value. Within the first cluster, selected individual GO terms related to influenza have also been displayed. (**b)** Graphical representation of the association between GO clusters. Nodes represent subclusters, with node size corresponding to the number of genes in that node. Edges represent association between subclusters in terms of biological processes, pathways, or PPI. GO analysis was conducted using the Metascape webtool and plotted in Cytoscape.

In order to identify common biological pathways, processes, and protein–protein interactions (PPI) within our list of putative restriction factors, a Gene Ontology (GO) analysis was conducted using Metascape (Fig. 7)^32^.

A total of n = 274 individual GO terms were significantly enriched with a significance threshold of *p* < 0.01. As expected, we see highly significant enrichment of terms related to influenza (Fig. 7a) including Transport of Virus (*p* = 2.0 × 10^−5^), Influenza Life Cycle (*p* = 5.8 × 10^−4^), and Influenza Infection (*p* = 7.7 × 10^−4^). While enrichment of influenza-related terms serves as a supporting additional validation of the screen results, the majority of the putative restriction factors identified are not associated with these ontology terms, suggesting analysis of other highly enriched GO terms may provide insight into novel antiviral pathways in the cell. Highly significant GO clusters, groups of related GO terms, are shown in Fig. 7a. Figure 7b provides a graphical representation of the interactions between subclusters. See Supplementary data file S6 for the full list of significantly enriched GO terms.

**Figure 8:**
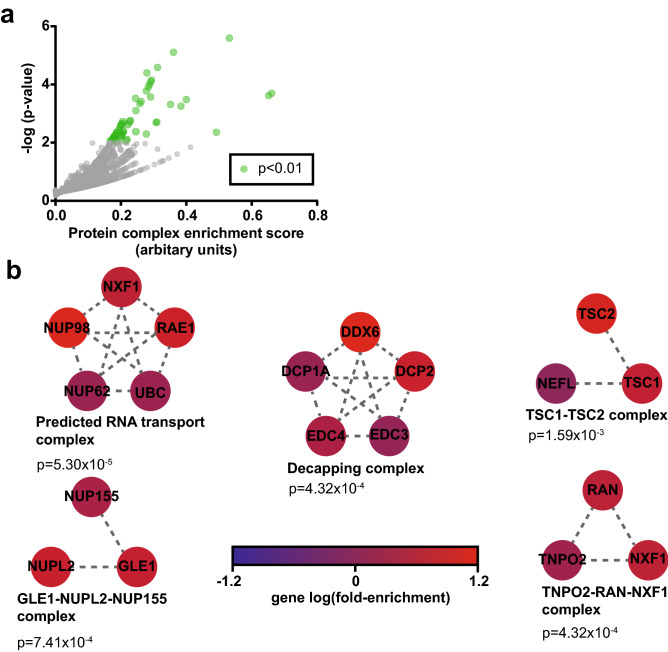
Protein complex enrichment analysis. (**a**) Significance and enrichment score (based on the COMPLEAT aggregate scoring system that factors in complex size, gene lfc, and other factors) of individual protein complexes. n = 84 protein complexes were found to be significantly enriched at a threshold of *p* < 0.01. (**b**) The top five enriched protein complexes identified, showing the individual genes corresponding to a given protein and lfc of that gene in the screen, as well as the interactions between proteins in the complex. Both *p* values and enrichment scores were generated using the COMPLEAT web tool.

In addition to the GO analysis, we also conducted an analysis for the enrichment of stable protein complexes using COMPLEAT^33^. If an individual restriction factor exerts its antiviral effect as part of a multiprotein complex, it follows that the knockout of any protein in that complex which is required for complex functionality should exert a similar antiviral effect. We would thus expect to see that some of the top ranked genes in the screen are part of the same protein heterocomplexes. This analysis is useful both as an indirect validation of the screen, and to guide subsequent efforts to create high yield knockout cell lines based on the screen results, as the knockout of multiple restriction factors that exert their effect through a common protein complex would be redundant. Results are summarized in Fig. 8.

As shown in Fig. 8a, n = 84 protein complexes (either described in literature or predicted through proteomics studies) were found to be significantly enriched with a threshold of *p* < 0.01. The highest scoring complexes as determined by the COMPLEAT algorithm are depicted as well in Fig. 8b. See Supplementary data file S7 for the full list of significantly enriched complexes.

## Discussion

In this work, we present a pooled screening strategy to identify and rank host restriction factors in vaccine-producing cell lines. In contrast to previous arrayed screens to identify host restriction factors in cell-based vaccine production systems, the use of a replication defective reporter virus and a pooled screen format allows genome-wide coverage without high-throughput robotics. The coculture of all cells in a given replicate also removes much of the inherent variability seen in large-scale arrayed screens^20^.

Using HEK-293SF cells and PR/8 influenza as a model, we identified 64 putative restriction factors that met the threshold for biological (lfc > 0.4) and statistical (*p* < 0.01) significance. The antiviral function of many of these restriction factors is implied from the fact that they are direct or indirect targets of influenza during infection; nuclear pore components like RAE1 and NUPL2, are downregulated by influenza during infection, which has been shown to benefit influenza replication^34^. Similarly, TSC1 and TSC2 negatively regulate the mTORC1 complex, which influenza has been shown to activate during late-stage replication to maximize viral protein production^35,36^. Other factors identified have known antiviral activity against influenza or similar viruses; DDX6 and DPC2 are components of the decapping complex, which has been shown to inhibit the expression of Bunyavirus mRNA by competing with the viral “cap snatching” pathway (similar to that utilized by influenza) for the 5′ cap of host mRNA^37^. SMG9 and UPF2 are a components of the nonsense mediated RNA decay (NMD) pathway, which normally functions to degrade aberrant transcripts with internal stop codons^38^. Internal stop codons are a common feature of viral transcripts, including those of influenza. Accordingly, NMD has been shown to exert antiviral effects on several RNA viruses^39^.

While antiviral function of many putative restriction factors identified in the screen is apparent, n = 41 of the factors identified have no association with a GO viral or antiviral process (see Supplemental S6). The results of this study therefore serve not only to guide the development of high yield knockout cell lines for influenza vaccine production, but also as a basis for studies into novel host antiviral pathways.

Interestingly, though by no means absent from the results, key mediators of canonical antiviral pathways, such as interferon and RIG-I, had a relatively minor impact on viral yield in this study. One explanation for this is that many vaccine production cell lines such as HEK-293, Vero, and MDCK show impaired antiviral signaling compared to primary cells, hence their inherent utility for virus production^12,15,40^. Another aspect to consider is that in vivo a major function of these pathways is to upregulate antigen presentation and recruit specialized immune cells, which is obviously not possible within in vitro cell culture systems^41,42^. Far more represented in the results are metabolic factors, particularly negative regulators of nucleic acid anabolism and translation (Fig. 7a). Even the highly represented non-canonical “antiviral” pathways identified, such as the decapping pathway, are activated not just in response to viral infection, but nutrient starvation as well. Together these results imply that in cell-based vaccine production systems, influenza yield is primarily determined by host cell metabolic state, rather than innate antiviral immunity, though there is significant overlap between these pathways.

The screening strategy presented here can in theory be applied to any cell-based vaccine production system. Even within the space of a single virus-host system, the FACS collection parameters can be adjusted depending on the end-use of the data. In this screen, for instance, we used a 1.5 drop yield mode and a high event rate during FACS acquisition, allowing a significant amount (~ 20%) of cells not in the 90th percentile of GFP expressing cells to contaminate the high yield fraction. In doing so, we sacrificed readout signal dynamic range to collect large numbers of cells and maintain library representation. This allowed us to generate a large list of hits for rich downstream bioinformatics analysis, while potentially compromising our ability to accurately rank those hits. Alternatively, if one was solely concerned with accurately ranking a smaller list of highly significant restriction factors for knockout cell line development, FACS acquisition could be run under more stringent conditions and with a lower fraction of cells collected (e.g. the 99th percentile of GFP expression). It should be noted though that even with the low dynamic range of the screen (± lfc1.2) the ranking of putative restriction factors was remarkably consistent between replicates; DDX6, for instance, was the top ranked gene in every replicate, and correlation between replicate samples was nearly 99%.

While the screen presented here is a useful tool for identifying and ranking viral restriction factors, it does have limitations; only restriction factors with an impact on viral protein expression can be identified, while those impacting viral packaging and egress are not. Furthermore, while this distinction is irrelevant for the purposes of increasing vaccine yield, there is no way to differentiate knockouts that induce a generic upregulation of translation from restriction factors that specifically inhibit viral replication, though this could be easily remedied with a stably expressed passive fluorescent reporter in host cells. There is also the possibility of false positive hits if a knockout somehow increases viral protein expression, but is detrimental to overall viral titer, as the results of Karlas et al. (2010) show is likely the case with NUP98^43^.

Overall the results show that the screening strategy we have demonstrated here is an effective and flexible tool to identify host restriction factors in cell-based influenza production systems. Future studies by our group will aim to exploit the results of this screen to generate high yield HEK-293SF knockout cell lines for influenza vaccine production, as well as elucidate host-virus interactions mediated by novel restriction factors identified in the screen.

## Methods

### Cell culture and maintenance

HEK-293SF cells (RRID accession: CVCL_4V94) were obtained from the National Research Council of Canada^22^. In all cases, cells were maintained in serum-free suspension at a density of 0.5–4.0 × 10^6^ in Hyclone Hycell TransFX-H media (GE Healthcare) supplemented with 4 mM Glutamine and 0.1% Pluronic F68 (Sigma-Aldrich). Cells were incubated in vented PETG shake flasks (Corning), spun at 110 rpm in a 37 °C incubator in a humidified 5% CO_2_ atmosphere. Cell counts and viability were determined by trypan blue exclusion assay using a Vi-CELL XR Cell Viability Analyzer (Beckman Coulter).

### Digital PCR (dPCR)

All dPCR assays were carried out on the QX200™ Droplet Digital™ PCR System (Bio-Rad) using the QX200 EvaGreen Digital PCR Supermix (Bio-Rad) according to the manufacturer’s instructions. Primers and thermocycling conditions for all dPCR assays are listed in Supplemental S8.

### Lentiviral vectors

Lentiviral vectors were produced in the HEK-293SF cell line as described previously^44^*.* The psPAX2 (Addgene #12260) and CMV-VSVG (Addgene #8454) plasmids were gifts from Didier Trono and Bob Weinberg, respectively^45^.

HEK-293SF cells were transduced with the lentiviral vectors by spinfection at 1,000 rcf for 45 min in media supplemented with 8 µg/mL of polybrene. Cells were then immediately resuspended in normal growth media to eliminate polybrene.

In all cases, infectious titer of lentiviral vectors was determined by dPCR assay, using a protocol adapted from Baczak et al. (2015)^46^. Briefly, HEK-293SF were transduced with serial tenfold dilutions of lentivirus. After 48 h, genomic DNA was extracted from cells using the Purelink Genomic DNA mini kit (Thermo Fisher) according to the manufacturer’s instructions. dPCR targeting the Woodchuck Hepatitis Virus Posttranscriptional Regulatory Element (WPRE) sequence of the vector genome was then used to determine the number of integrated vector genomes/cell. A parallel dPCR assay targeting the albumin gene was used as a normalization control. See Supplemental File S8 for primers and thermocycling conditions.

### A/Puerto Rico/8/1934 influenza

PR/8 influenza (NCBI txid:211,044) stocks were generated by reverse genetics in HEK-293SF cells. The process was described previously in Milián et al. (2017)^9^. The reverse genetics constructs used were a generous gift from Xuguang Li’s group at Health Canada, and their construction was described previously in Neumann et al. (2005)^47^.

To determine infectious particle titer, tenfold dilutions of virus were used to infect HEK-293SF cells. After a 3-h incubation, cells were stained for influenza NP expression and the percent of cells expressing influenza NP quantified by flow cytometry. See Sect. Flow cytometry and FACS for details on staining and flow cytometry. Only cultures showing between 2 and 20% of cells infected were used for quantification to minimize error due to superinfection.

Viral genomes were quantified by extracting RNA from cell-free supernatants using the QIAamp Viral RNA Mini Kit (Qiagen) according to the manufacturer’s instructions. RNA was then reverse transcribed with the iScript Select Reverse Transcription Kit (Bio-Rad) according to the manufacturer’s instructions and using gene specific primers targeted to influenza genomic segment 7(M). dPCR assay using the same primers was then used to determine viral genome copy number. See Supplemental File S8 for primers and thermocycling conditions.

All PR/8 infections were carried out at an MOI of 0.1 at a cell density of 10^6^ cells/ml. PR/8 cultures were supplemented with 1 µg/mL of 6-(1-tosylamido-2-phenyl) ethyl chloromethyl ketone (TPCK) trypsin (Sigma-Aldrich) to allow proteolytic activation of HA.

### PR/8^GFPΔHA^ reporter influenza

The PR/8^GFPΔHA^ virus was a generous gift from Alain Townsend (Oxford University). The cloning and production of this virus was previously described in Powell et al. (2012)^48^. Briefly, the coding sequence of the HA gene is removed and replaced with that of GFP. The virus is then propagated in an HA-expressing MDCK line. All PR/8^GFPΔHA^ infections were carried out at a multiplicity of infection (MOI) of 5.

### Knockout pool generation

To generate the HEK-293SF^ΔTBK1^ knockout pool, an sgRNA was designed using CHOPCHOP (v.2)^49^ to target the first exon of the TBK1 gene (NCBI accession:NG_046906.1). sgRNA for the HEK-293SF^ΔDDX6^, HEK-293SF^ΔSMG9^, and HEK-293SF^ΔCARM1^ knockout pools were randomly selected from corresponding Brunello library guides for that gene^21^. This sgRNA was then cloned into LentiCRISPR.V2 (Addgene #52961) using standard techniques and verified by Sanger sequencing. LentiCRISPR.V2 was a gift from Feng Zhang^50^. Lentiviral vectors produced using this construct were used to infect HEK-293SF at an MOI of 10. Following selection with 2 µg/mL Puromycin for 48 h, CRISPR editing efficiency was assessed using the TIDE webtool (v2.0.1)^51^. Primers used to generate PCR amplicons for this analysis are listed in Supplemental S8. Cells were then incubated for a further 10 days to allow knockout phenotypes to manifest and recover the drop in cell viability arising from DNA cleavage. TBK1 knockout was further verified by Western blot using mouse αTBK1 (Santa Cruz, sc9085) at a 1/200 dilution. αβ-Actin (Sigma A1978) at a 1/1,000 dilution was used as a loading control.

### Brunello library knockout pool generation

The Human Brunello CRISPR knockout pooled library was a gift from David Root and John Doench (Addgene #73178)^21^. Lentiviral preparations obtained from Addgene were used to transduce cells at an MOI of 2, incubated for 48 h, and then selected with 2 µg/mL Puromycin for 48 h. Cells were then incubated for a further 10 days to allow knockout phenotypes to manifest and recover the drop in cell viability arising from DNA cleavage. Aliquots of cells were then frozen in 10% DMSO. Cells were thawed and subcultured for 48 h before use in the screen. At all points, a minimum representation of 300 copies/sgRNA was maintained.

### Flow cytometry and FACS

For each of the three screen replicates, cells were sorted live, and sorting was carried out on the FACSJazz (BD Biosciences) instrument using 1.5 drop yield mode and an event rate of 7,000–9,000 events/second. Cells were sorted 33–40 hpi with PR/8^GFPΔHA^, a window in which GFP expression was stable, but cell viability was still high (Figs. 2, 3). Two fractions of cells were collected: the top 10% of GFP expressing cells (“high yield”), and a control fraction consisting of all infected cells (i.e. GFP positive). For both fractions, the number of cells collected is such that a minimum representation of 300 copies/sgRNA was maintained. Flow cytometry was carried out on the Accuri C6 (BD Biosciences) instrument. GFP-expressing samples were run live. In cases where influenza NP protein expression was assessed, cells were fixed and permeabilized with the BD Transcription Factor Buffer Set (BD Pharmingen). Infected cells were then identified by staining with a 1:50 dilution of mouse αNP-FITC (Thermo Fisher, clone D67J) for 50 min. In cases where GFP expression interfered with the use of the conjugated FITC fluorophore, goat αmouse-PE-Cy5.5 (Thermo Fisher, cat#M32218) was used as a secondary antibody, and any GFP/FITC fluorescence compensated for. In all cases, data analysis was conducted using FlowJo (v.10).

### Deep sequencing sample preparation and sequencing

gDNA was extracted from cells using the JetQuick Blood and Cell Culture DNA Midiprep Kit (Thermo Fisher). PCR was then used to amplify the sgRNA inserts and append Illumina adaptors and hexamer barcodes to the amplicons. See Supplemental S8 for primer sequences and thermocycling. PCR was performed using the Q5 Hot Start High-Fidelity 2X Master Mix (New England Biolabs). Before creating the amplicon library, dPCR was used to assay the copy number of sgRNA inserts in extracted genomic DNA. Sufficient genomic DNA template was used to ensure a minimum read depth of 300 per sample. PCR products were then pooled, concentrated by isopropanol precipitation, and gel purified on a 2% agarose gel before sequencing. Gel extraction was carried out with the PureLink Gel Extraction kit (Thermo Fisher). The purified, barcoded amplicon libraries were then pooled and sequenced as single-read 50 bp reads on the Illumina HiSeq 4,000 (Illumina).

### Bioinformatics

Data to assess sequencing quality and read mapping, as well as quantification of the changes in sgRNA abundance between the high yield and control cell populations was carried out using the MAGeCK software suite (v.0.5.9.2)^31,52,53^. In this analysis, read counts were normalized using a set of 1,000 nontargeting control sgRNA’s that were provided in the Brunello library. The initial set of 1,000 was reduced to 963 after the removal of outliers whose difference in normalized read counts between conditions were outside of the range [Q1 − 1.5 ∗ IQR, Q3 + 1.5 ∗ IQR], where Q1, Q3, and IQR are the first quartile, third quartile, and interquartile range, respectively. To test for significance of sgRNA abundance between conditions, the MAGeCK “tool” test was used with additional parameters –remove-zero and –remove-zero-threshold set to "control" and "30 ", respectively. This removed 1,760 sgRNA’s that have an average read count in the control condition that is less than 30. All other parameters were left at the default setting. Of note, sgRNA-level *p* values were adjusted using the Benjamini–Hochberg procedure, which controls the False Discovery Rate at level α = 0.25. To obtain gene-level *p* values from multiple sgRNA’s targeting a single gene, version 0.5.9 of the modified Robust Rank Aggregation (RRA) algorithm, named α-RRA, was used^54^.

GO analysis was carried out using the Metascape webtool (v.3.5), and plotted using default parameters^32^. Plots were created using Cytoscape (v.3.7.2)^55^. Protein complex enrichment analysis was carried out using the COMPLEAT webtool (v.1.0)^33^. Genes were submitted as a single list using lfc as a ranking metric.

### Statistical analysis

Where stated, the coefficient of correlation and statistically significant differences between two groups of means were determined by Student’s *t* test using Prism GraphPad (v.6.01). Error bars in figures represent SEM.

## Supplementary information

Supplementary information S1.

Supplementary information S2.

Supplementary information S3.

Supplementary information S4.

Supplementary information S5.

Supplementary information S6.

Supplementary information S7.

Supplementary information S8.

## Data Availability

All relevant data supporting the findings of this study are contained within the paper and supplementary information files, with the exception of the raw FASTQ sequencing files. Raw FASTQ data for screen replicates can be accessed by reasonable request to the corresponding author.

## Code availability

This study did not make use of custom code. The MAGeCKFlute software suite is freely available via the following link: https://bioconductor.org/packages/release/bioc/html/MAGeCKFlute.html.
