# Supplementary material for: A pooled genome-wide screening strategy to identify and rank influenza host restriction factors in cell-based vaccine production platforms
Source: Sci Rep. 2020 Jul 22;10:12166. doi: 10.1038/s41598-020-68934-y (PMC7376217; doi:10.1038/s41598-020-68934-y)

**A pooled genome-wide screening strategy to identify and rank influenza host restriction factors in cell-based vaccine production platforms**

David M. Sharon, Sean Nesdoly, Hsin J. Yang, Jean-François Gélinas, Yu Xia, Sven Ansorge, Amine A. Kamen*

**Supplemental S2. FACS gating strategies**

In all flow cytometry and FACS applications in this study, the first two gating steps were the same. **a)** whole cells are isolated from debris and large clumps followed by **b)** doublet discrimination based on pulse width. For cell sorting during the screen, two populations of cells were collected: **c)** a high yield fraction consisting of the top 10% of GFP expressing cells and **d)** a control fraction consisting of all infected (GFP positive) cells. **e)** For the purposes of titrating the PR/8GFPΔHA reporter virus, infected cells were compared to a mock infected control, with the threshold for GFP expression set at 1% of the control. **f)** For the purposes of titrating and monitoring wildtype PR/8 infection kinetics by means of influenza NP expression, infected cells were compared to a mock infected control, with the threshold for GFP expression set at 1% of the control.

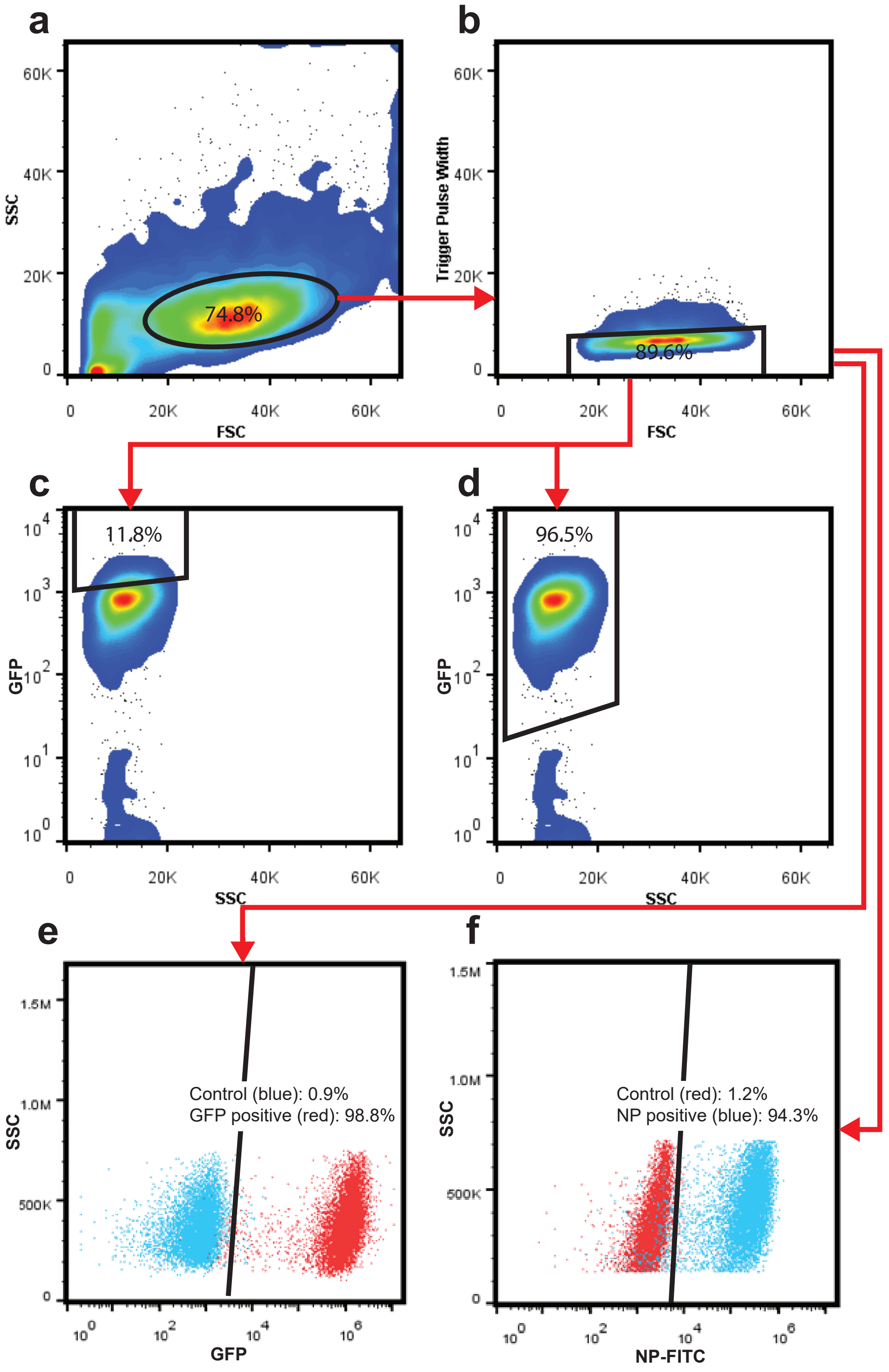

Supplement: Supplementary file 2 — Supplementary information S2. [file 41598_2020_68934_MOESM2_ESM.pdf]
