# Supplementary material for: A pooled genome-wide screening strategy to identify and rank influenza host restriction factors in cell-based vaccine production platforms
Source: Sci Rep. 2020 Jul 22;10:12166. doi: 10.1038/s41598-020-68934-y (PMC7376217; doi:10.1038/s41598-020-68934-y)

A pooled genome-wide screening strategy to identify and rank influenza host restriction factors in cell-based vaccine production platforms

David M. Sharon, Sean Nesdoly, Hsin J. Yang, Jean-François Gélinas, Yu Xia, Sven Ansorge, Amine A. Kamen*

Supplemental S3. TBK1 Western blot

a) αTBK1 blot **b)** αBeta-Actin blot. Third lane on gel is another TBK1 knockout pool that was not used in this study.

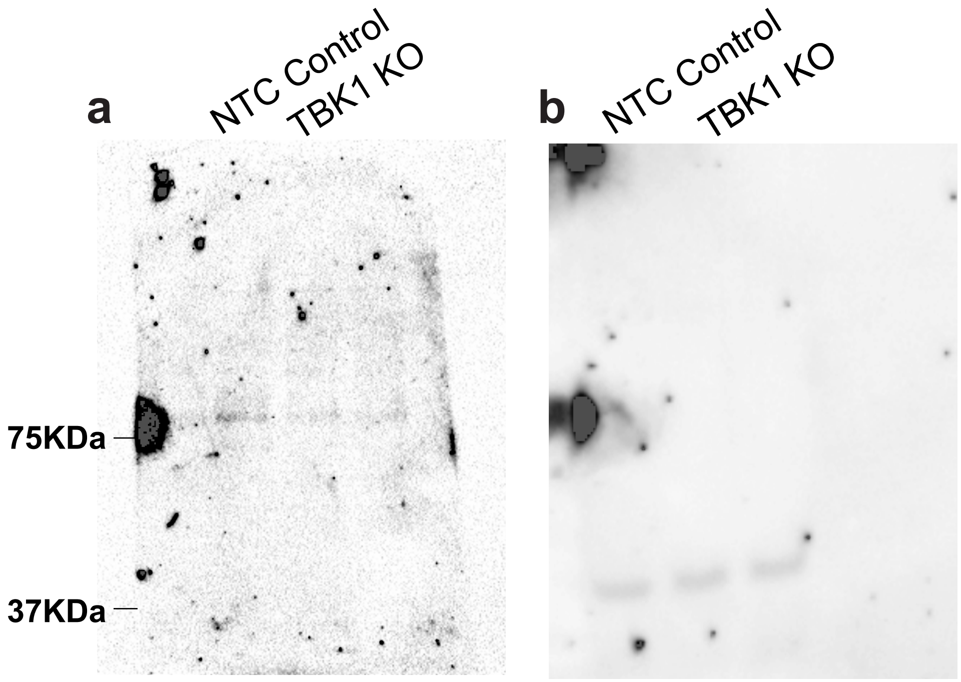

Supplement: Supplementary file 3 — Supplementary information S3. [file 41598_2020_68934_MOESM3_ESM.pdf]
