# Supplementary material for: A pooled genome-wide screening strategy to identify and rank influenza host restriction factors in cell-based vaccine production platforms
Source: Sci Rep. 2020 Jul 22;10:12166. doi: 10.1038/s41598-020-68934-y (PMC7376217; doi:10.1038/s41598-020-68934-y)

A pooled genome-wide screening strategy to identify and rank influenza host restriction factors in cell-based vaccine production platforms

David M. Sharon, Sean Nesdoly, Hsin J. Yang, Jean-François Gélinas, Yu Xia, Sven Ansorge, Amine A. Kamen*

Supplemental S5. TIDE analysis of DDX6, SMG9, and CARM1 knockout pools

TIDE analysis was used to assess CRISPR editing efficiency in the **a)** DDX6 **b)** SMG9, and **c)** CARM1 knockout pools. Results indicate editing efficiencies of >80% in all cases.

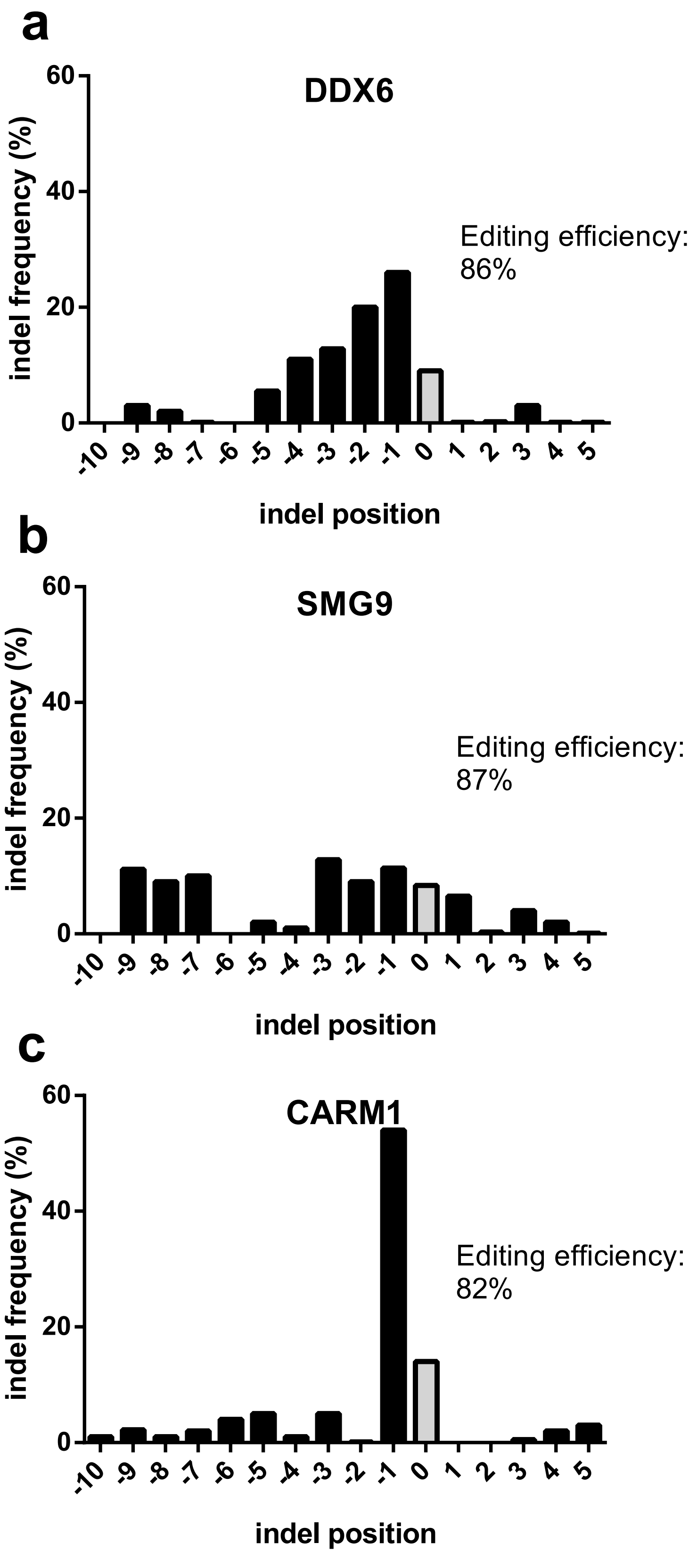

Supplement: Supplementary file 5 — Supplementary information S5. [file 41598_2020_68934_MOESM5_ESM.pdf]
