# Supplementary material for: A pooled genome-wide screening strategy to identify and rank influenza host restriction factors in cell-based vaccine production platforms
Source: Sci Rep. 2020 Jul 22;10:12166. doi: 10.1038/s41598-020-68934-y (PMC7376217; doi:10.1038/s41598-020-68934-y)
Supplement: Supplementary file 8 — Supplementary information S8. [file 41598_2020_68934_MOESM8_ESM.pdf]

A pooled genome-wide screening strategy to identify and rank influenza host restriction factors in cell-based vaccine production platforms

David M. Sharon, Sean Nesdoly, Hsin J. Yang, Jean-François Gélinas, Yu Xia, Sven Ansorge, Amine A. Kamen

Supplemental S8. Primers and thermocycling conditions

dPCR assays

For measurement of total influenza viral particles in CFS

Influenza A segment 7, forward	GACCRATCCTGTCACCTCTGAC
Influenza A segment 7, reverse	AGGGCATTYTGGACAAAKCGTCTA

1) 95°C, 10min 2) 95°C, 30s 3) 61°C, 30s 4) 72°C, 30s 5) Back to step 2, 39 cycles 6) 72°C, 10min 7) 4°C, hold

For measurement of integrated lentiviral vector copy number

WPRE, forward	GTCCTTTCCATGGCTGCTC
WPRE, reverse	CCGAAGGGACGTAGCAGA
Albumin, forward	TTTGCAGATGTCAGTGAAAGAGA
Albumin, reverse	TGGGGAGGCTATAGAAAATAAGG

1) 95°C, 10min 2) 95°C, 30s 3) 60°C, 30s 4) Back to step 2, 39 cycles 5) 72°C, 10min 6) 4°C, hold

For measurement of influenza segment 4 mRNA (GFP and HA)

GFP, forward	CTGCTGCCCGACAACCAC
GFP, reverse*	TCACGAACTCCAGCAGGAC
HA, forward	ATCGACTATGAGGAGCTGAGGG
HA, reverse*	GCCGTTACTCCGTTTGTGTTGT
Actin, forward	GTCATACTCCTGCTTGCTGAT
Actin, Reverse	AAAGACCTGTACGCCAACAC

*Reverse transcription was carried out using these primers as gene-specific primers to selectively amplify positive-sense RNA

1) 95°C, 10min 2) 95°C, 30s 3) 60°C, 30s 4) Back to step 2, 39 cycles 5) 72°C, 10min 6) 4°C, hold

<u>Knockout pools</u>

TBK1

sgRNA sequence	TTCCGCGGCCACGGTAATGA
TIDE assay primer, forward	GGCCGTTTTCCAAAATACCGA
TIDE assay primer, reverse	GATGCAGGTCGAGGACCG

1) 95ºC, 60s 2) 95ºC, 15s 3) 63ºC, 15s 4) Back to step 2, 28 cycles 5) 72ºC, 5min 6) 4ºC, hold

DDX6

sgRNA sequence	AGGTCTAGCCGTTCAAGTAA
TIDE assay primer, forward	TGTTGCAGGGATGAGGTGTC
TIDE assay primer, reverse	CCTGTCTCACTGGAATGCTGT

1) 98ºC, 3min 2) 98ºC, 30s 3) 63ºC, 30s 4) 72ºC, 60s 5) Back to step 2, 34 cycles 6) 72ºC, 5min 7) 4ºC, hold

SMG9

sgRNA sequence	GCTGAAATGAAGGAACGAGG
TIDE assay primer, forward	TCAAAACATGCACTACCCCC
TIDE assay primer, reverse	CCAGTCAGTGCTAACGACAGT

1) 98ºC, 3min 2) 98ºC, 30s 3) 63ºC, 30s 4) 72ºC, 60s 5) Back to step 2, 34 cycles 6) 72ºC, 5min 7) 4ºC, hold

CARM1

sgRNA sequence	TCGCGTCGCCGATGGTGAGG
TIDE assay primer, forward	TTGTGTGGGGCGGGGTA
TIDE assay primer, reverse	GCTCCCTTGCTCACTCTGG

1) 98ºC, 3min 2) 98ºC, 30s 3) 63ºC, 30s 4) 72ºC, 60s 5) Back to step 2, 34 cycles 6) 72ºC, 5min 7) 4ºC, hold *Use high GC buffer

Non-targeting control (NTC)

sgRNA sequence	TTCCGCGGCCACGGTAATGA

Illumina amplicon library prep/barcoding

P5_0nt_stagger*	AATGATACGGCGACCACCGAGATCTACACTCTTTCCCTACACGAC GCTCTTCCGATCTTTGTGGAAAGGACGAAACACCG
P5_1nt_stagger*	AATGATACGGCGACCACCGAGATCTACACTCTTTCCCTACACGAC GCTCTTCCGATCTCTTGTGGAAAGGACGAAACACCG
P5_2nt_stagger*	AATGATACGGCGACCACCGAGATCTACACTCTTTCCCTACACGAC GCTCTTCCGATCTGCTTGTGGAAAGGACGAAACACCG
P5_3nt_stagger*	AATGATACGGCGACCACCGAGATCTACACTCTTTCCCTACACGAC GCTCTTCCGATCTAGCTTGTGGAAAGGACGAAACACCG
P5_4nt_stagger*	AATGATACGGCGACCACCGAGATCTACACTCTTTCCCTACACGAC GCTCTTCCGATCTCAACTTGTGGAAAGGACGAAACACCG
P5_6nt_stagger*	AATGATACGGCGACCACCGAGATCTACACTCTTTCCCTACACGAC GCTCTTCCGATCTTGCACCTTGTGGAAAGGACGAAACACCG
P5_7nt_stagger*	AATGATACGGCGACCACCGAGATCTACACTCTTTCCCTACACGAC GCTCTTCCGATCTACGCAACTTGTGGAAAGGACGAAACACCG
P5_8nt_stagger*	AATGATACGGCGACCACCGAGATCTACACTCTTTCCCTACACGAC GCTCTTCCGATCTGAAGACCCTTGTGGAAAGGACGAAACACCG
P7_Barcode1	CAAGCAGAAGACGGCATACGAGATCGAGTAGTGACTGGAGTTCA GACGTGTGCTCTTCCGATCTCCAATTCCCACTCCTTTCAAGACCT
P7_Barcode2	CAAGCAGAAGACGGCATACGAGATTCTCCGGTGACTGGAGTTCA GACGTGTGCTCTTCCGATCTCCAATTCCCACTCCTTTCAAGACCT
P7_Barcode3	CAAGCAGAAGACGGCATACGAGATAATGAGGTGACTGGAGTTCA GACGTGTGCTCTTCCGATCTCCAATTCCCACTCCTTTCAAGACCT
P7_Barcode4	CAAGCAGAAGACGGCATACGAGATGGAATCGTGACTGGAGTTCA GACGTGTGCTCTTCCGATCTCCAATTCCCACTCCTTTCAAGACCT
P7_Barcode5	CAAGCAGAAGACGGCATACGAGATTTCTGAGTGACTGGAGTTCA GACGTGTGCTCTTCCGATCTCCAATTCCCACTCCTTTCAAGACCT
P7_Barcode6	CAAGCAGAAGACGGCATACGAGATACGAATGTGACTGGAGTTCA GACGTGTGCTCTTCCGATCTCCAATTCCCACTCCTTTCAAGACCT
Illumina adaptor - Stagger nucleotides - Index sequence - Vector binding sequence	

*The eight P5 primers were pooled for use in barcoding PCR

1) 95°C, 60s 2) 95°C, 30s 3) 53°C, 30s 4) 72°C, 30s 5) Back to step 2, 28 cycles 6) 72°C, 10min 7) 4°C, hold
